# Nanosized Zinc Oxide Induces Toxicity in Human Lung Cells

**DOI:** 10.1155/2013/316075

**Published:** 2013-08-07

**Authors:** Devashri Sahu, G. M. Kannan, R. Vijayaraghavan, T. Anand, Farhath Khanum

**Affiliations:** ^1^Pharmacology and Toxicology Division, Defence Research and Development Establishment, Jhansi Road, Gwalior 474001, India; ^2^Saveetha University, Chennai 600077, India; ^3^Biochemistry and Nutrition Discipline, Defence Food Research Laboratory, Mysore 570011, India

## Abstract

Zinc oxide nanoparticles (ZnO-NPs) are increasingly used in sunscreens, biosensors, food additives, pigments, rubber manufacture, and electronic materials. With the wide application of ZnO-NPs, concern has been raised about its unintentional health and environmental impacts. This study investigates the toxic effects of ZnO-NPs in human lung cells. In order to assess toxicity, human lung epithelial cells (L-132) were exposed to dispersion of 50 nm ZnO-NPs at concentrations of 5, 25, 50, and 100 **μ**g/mL for 24 h. The toxicity was evaluated by observing changes in cell morphology, cell viability, oxidative stress parameters, DNA damage analysis, and gene expression. Exposure to 50 nm ZnO-NPs at concentrations between 5 and 100 **μ**g/mL decreased cell viability in a concentration-dependent manner. Morphological examination revealed cell shrinkage, nuclear condensation, and formation of apoptotic bodies. The oxidative stress parameters revealed significant depletion of GSH level and increase in ROS levels suggesting generation of oxidative stress. ZnO-NPs exposure caused DNA fragmentation demonstrating apoptotic type of cell death. ZnO-NPs increased the expression of metallothionein gene, which is considered as a biomarker in metal-induced toxicity. To summarize, ZnO-NPs cause toxicity in human lung cells possibly through oxidative stress-induced apoptosis.

## 1. Introduction

Over the past decade the ability to engineer and produce materials at the nano- or near-atomic scale has triggered rapid product development due to their new interesting properties that were not previously seen at scales above the micrometer. Industrial applications using nanoparticles have resulted in an almost exponentially growing demand for nanosized materials. Due to increasing use of nanoparticles in variety of consumer goods, humans are constantly exposed to such nanomaterials besides exposure at production sites [1–5]. Unintended exposure to nanomaterials may occur via inhalation, dermal exposure, or gastrointestinal tract absorption and may pose a great risk [6, 7]. Despite their wide application, little is known about their effect on human health and environment.

Zinc oxide (ZnO) is among the most commonly utilized group of nanomaterials and has wide-ranging applications [8]. As a well-known photocatalyst, ZnO has received much attention in the degradation and complete mineralization of environmental pollutants [9, 10]. ZnO nanoparticles (ZnO-NPs) are used in industrial products including cosmetics, paints, and medical materials. ZnO-NPs have external uses as antibacterial agents in ointments, lotions, mouthwashes, and surface coatings to prevent microorganism growth [11]. ZnO-NPs have also been used as a dietary supplement in human and livestock because zinc can stimulate the immune system and act in an anti-inflammatory way [12, 13]. 

Many *in vitro* studies demonstrated that ZnO-NPs are toxic to mammalian cells and are even more toxic than other nanoscale structures of metallic oxide [14–16]. In combination with UV exposure, ZnO-NPs are known to generate reactive oxygen species (ROS) like hydroxyl radicals or hydrogen peroxide in aqueous solutions leading to efficient decomposition of organic compounds [17]. Brunner et al. [18] showed that a three-day exposure of human mesothelioma and rodent fibroblast cell to ZnO-NPs (19 nm) caused DNA and mitochondrial damages. 

The human lung is a vulnerable organ for nanoparticle invasion as there is approximately 2300 km of airways and 300 million alveoli, giving rise to a large surface area, which is in contact with the environmental atmosphere and the ultrafine particulate pollutants present in it [19, 20]. Adverse systemic reactions have been observed after inhalation of ZnO fumes or accidental ingestion of large amounts of the metal [21–23]. Inhalation of ZnO has been found to compromise pulmonary function in pigs and caused pulmonary impairment and metal fume fever in humans [24, 25]. Karlsson et al. [26] found that ZnO-NPs (71 nm) decreased cell viability and caused oxidative DNA damage in human alveolar carcinoma epithelial cells (A549). Despite the wide inhalational exposure of ZnO-NPs, there are very few studies to demonstrate its toxicity on normal human lung cells and the possible mechanism of toxicity.

Therefore, the present study evaluated the toxicity of 50 nm ZnO-NPs in human lung epithelial cells (L-132) and tried to elucidate involvement of oxidative stress therein. As zinc-mediated metallothionein (MT) upregulation has been found to protect against oxidative stress-induced cellular injury [27], attempts were also made to study the effect of ZnO-NPs on expression of MT in L-132 cells.

## 2. Materials and Methods

### 2.1. Chemicals

Dulbecco's modified eagle's medium (DMEM), penicillin, streptomycin, fetal bovine serum (FBS), 3-(4,5-dimethylthiazol-2-yl)-2,5-diphenyltetrazolium bromide (MTT), 2,7-dichlorofluorescin diacetate (DCFH-DA), metaphosphoric acid (MPA), dimethyl sulfoxide (DMSO), *o*-phthaldialdehyde (OPT), and 5,5′-dithiobis(2-nitrobenzoic acid) were purchased from Sigma Chemical Company (St. Louis, MO, USA). Leishman's stain solution was purchased from Fisher scientific. The ZnO-NPs (50 nm) used in this study were synthesized in DRDE (Defence Research and Development Establishment, Gwalior, India), by sol gel method [28]. 

### 2.2. Cell Culture

The human lung epithelial cell line (L-132) was purchased from National Centre of Cell Science (NCCS, Pune, India). Cells were cultured in DMEM medium containing 10% FBS and 100 U/mL penicillin-streptomycin-amphotericin mixture and incubated at 37°C with 5% CO_2_.

### 2.3. Characterization of Nanoparticle

Physicochemical properties of particles were analyzed using transmission electron microscopy (TEM), dynamic light scattering (DLS), and zeta potential analyzer. The morphology and size of nanoparticles in the stock dispersion were determined by transmission electron microscopy (TEM). Dry powder of particles was suspended in cell culture medium at a concentration of 1 mg/mL and then sonicated at room temperature for 10 min at 40 W to form a homogeneous suspension. After sonication and stabilization, the TEM samples were prepared by drop coating of the stock suspension on carbon-coated copper grids. The films on the grids were allowed to dry prior to measurement. TEM measurements were performed at an accelerating voltage of 120 kV (Model 1200EX, JEOL Ltd., Tokyo, Japan). ZetaPALS (Brookhaven Instruments Corporation, Holtsville, NY) was used to determine the hydrodynamic size and zeta potential of particle suspension in cell culture medium.

### 2.4. ZnO-NPs Exposure

The sonicated dispersion of ZnO-NPs was used to expose the cells at 5, 25, 50, and 100 *μ*g/mL for 24 h. Based on the results of previous study done (data not shown) with different doses of nanoparticles, a dose range was selected which is also studied by Ahamed et al. [29]. Exposure of cells was performed with 80% confluence of cell in 25 cm^2^ flasks and 24-well plates in a humidified atmosphere at 37°C and 5% CO_2_. Cells free of ZnO-NPs were used as control cells throughout each assay. 

### 2.5. Assessment of Cytotoxicity

L-132 cells were plated into a 24-well plate at a density of 50,000 cells/well. Cells were grown overnight in the full medium followed by exposure to ZnO-NP. Following the exposure of 24 h, cell viability was measured by MTT assay [30]. The cells were incubated with MTT (5 mg/mL) for 4 h. The medium was then removed, and 200 *μ*L of DMSO was added into each well to dissolve formazan crystals, the metabolite of MTT. After thorough mixing, the plate was read at 570 nm for optical density that directly correlates with cell quantity. Survival rate was calculated from the relative absorbance at 570 nm and expressed as the percentage of control. 

### 2.6. Qualitative Observation of Cellular Morphology

Cells were plated into a 6-well culture plate at a density of 75000 cells/well (in 2 mL growth medium). After overnight growth, supernatants from the culture plates were aspirated out, and fresh aliquots of growth medium containing ZnO-NPs in desired concentrations (5–100 *μ*g/mL) were added. Upon incubation, cells were washed with phosphate buffered saline (0.1 M PBS, pH 7.4), and the morphological changes were observed using Leishman's stain under an inverted phase contrast microscope at 200x magnification.

### 2.7. Intracellular Reactive Oxygen Species Measurement

The production of intracellular reactive oxygen species (ROS) was measured using DCFH-DA [31]. DCFH-DA passively enters the cell, where it reacts with ROS to form a highly fluorescent compound, dichlorofluorescein (DCF). L-132 cells were plated into a 24-well plate at a density of 50,000 cells/well. The cells were incubated at 37°C for 30 min with DCFH-DA working solution (100 *μ*M in methanol) to yield a 10 *μ*M into each well. Then cells were washed twice with PBS and exposed to different concentrations of ZnO-NP for 6, 12, and 24 h. After exposure to ZnO-NP, the fluorescence was observed at 485 nm excitation and 525 nm emission using a Bio-Tek Synergy HT-I plate reader (Bio-Tek Instruments, USA).

### 2.8. Measurement of Intracellular Reduced Glutathione (GSH) Level

Cellular levels of GSH were determined using Hissin and Hilf method [32]. The method is based on a reaction between GSH and *o*-phthaldialdehyde (OPT) which gives the fluorescence. Thus GSH concentration in a sample solution can be determined by observing the fluorescence at 360 nm excitation and 420 nm emissions. L-132 cells were seeded into a 25 cm^2^ flask at a density of 1.0 × 10^5^ cells. After 24 h exposure to ZnO-NPs, the cells were scraped and pelleted by centrifugation at 5000 rpm for 5 min, then washed in PBS. The cells were homogenized in 200 *μ*L of phosphate-EDTA buffer pH 8.0 and 80 *μ*L of 20% metaphosphoric acid. The cell homogenate was centrifuged at 16000 rpm at 4°C for 30 min. The assay was performed by taking 100 *μ*L supernatants and mixing it with 800 *μ*L of phosphate-EDTA buffer containing 100 *μ*L OPT (10 mg/mL in methanol). After thorough mixing and incubation at room temperature for 15 min, fluorescence was measured at 360 nm excitation and 420 nm emission using Bio-Tek Synergy HT-I plate reader (Bio-Tek Instruments, USA). Results were calculated as nmol of glutathione per mg of protein and presented as a percentage of the control group. Protein assays in the cell lysate were performed using a BCA protein assay kit (Pierce, USA).

### 2.9. Detection of DNA Damage

L-132 cells (5 × 10^5^ cells) exposed to ZnO-NPs (5, 25, 50, and 100 *μ*g/mL) for 48 h were collected into tubes and washed with PBS. The cells were incubated for 3 h in lysis buffer (20 mM Tris-HCl, pH 8.0, 5 mM EDTA, 0.1 M NaCl, 0.5% SDS, and 100 *μ*g/mL RNase) at 37°C. After incubation, phenol : chloroform (1 : 1) mixture was used to extract DNA. By adding an equal volume of ice-cold absolute isopropanol, DNA was precipitated. DNA was dissolved in 50 *μ*L of 1X TE (10 mM Tris, 1 mM EDTA, pH 8.0). Twenty  *μ*g of DNA was loaded onto 1.5% agarose gel; electrophoresis was carried out at 60 V for 2 h with TBE as the running buffer. DNA in the gel was visualized under UV light [33]. 

### 2.10. Hoechst Staining

The fluorescent probe Hoechst 33342 stains nuclei specifically, with little or no cytoplasm labeling. Cells exposed to different concentrations of ZnO-NPs (50 nm) were collected and sequentially washed by PBS. Then, the cells were kept in 1 *μ*g/mL Hoechst working solution for 15 min in the dark at room temperature [34]. Finally, the cells were washed twice with PBS to remove excess stain and examined under fluorescent microscope (Leica-DMLB, Germany) with excitation wavelength of 403 nm. 

### 2.11. Gene Expression Analysis

Human metallothionein (MT) messenger RNA (mRNA) expression was determined by reverse transcriptase polymerase chain reaction (RT-PCR). Cells were exposed to 5, 25, 50, and 100 *μ*g/mL of ZnO-NP for 24 h, and total RNA was isolated using RNeasy Mini Kit (Qiagen, USA). The concentration and integrity of RNA were measured using nanodrop spectrophotometer prior to the experiment. The Enhanced Avian HS RT-PCR Kit (Sigma, USA) was used for the amplification of human MT-1 and glyceraldehyde-3-phosphate dehydrogenase (GAPDH) gene, according to the manufacturer's instructions. Amplified cDNA products were separated on 1.2% agarose gel by electrophoresis. The sequences of primer sets used were MT-1 forward, 5′-CCACTGCTTCTTCGCTTCTC-3′ and reverse, 5′-AGGAGCAGCAGCTCTTCTTG-3′; GAPDH forward, 5′-GGCGCTGAGTACGTCGTGGAGT-3′ and reverse 5′-CGCCTGCTTCACCACCTTCTTG-3′.

### 2.12. Statistics

All data were reported as mean ± standard deviation (SD). Statistical analysis was performed for the experiments conducted in at least triplicate using one-way ANOVA followed by Dunnett test. Results with *P* < 0.05 were considered as statistically significant. 

## 3. Results

### 3.1. Particle Characterization

The average size reported by TEM was 50.24 ± 8.19 nm. Other physicochemical characteristics are detailed in Table 1. 

### 3.2. Concentration-Dependent Cytotoxicity of ZnO-NPs

**Figure 1:**
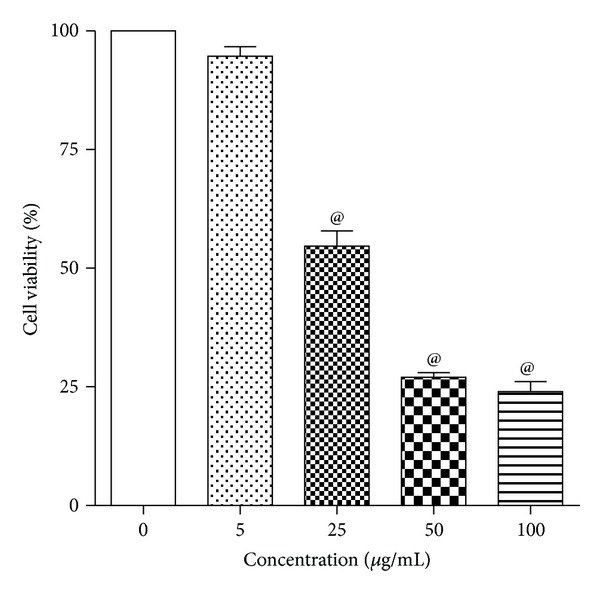
Concentration-dependent toxicity of ZnO-NPs in L-132 cells. Cells were exposed to different concentrations of ZnO-NPs particles for 24 h, and the viability was determined by MTT assay. Control cells cultured in particle-free medium were run in parallel to the exposed groups. Values were the mean ± SD from three independent experiments. Significance was indicated by ^@^ *P* < 0.05 versus control.

MTT assay showed that exposure of human lung epithelial cells (L-132) to 50 nm ZnO-NPs at concentrations of 25, 50, and 100 *μ*g/mL for 24 h significantly reduced the cell viability in a concentration-dependent manner. However, reduction in cell viability at lower concentration of 5 *μ*g/mL was not significant. As the concentrations of ZnO-NPs increased from 25 to 100 *μ*g/mL, the cell viability significantly decreased from 55% to 25% (Figure 1).

### 3.3. Effect of ZnO-NP on Cellular Morphology

**Figure 2:**
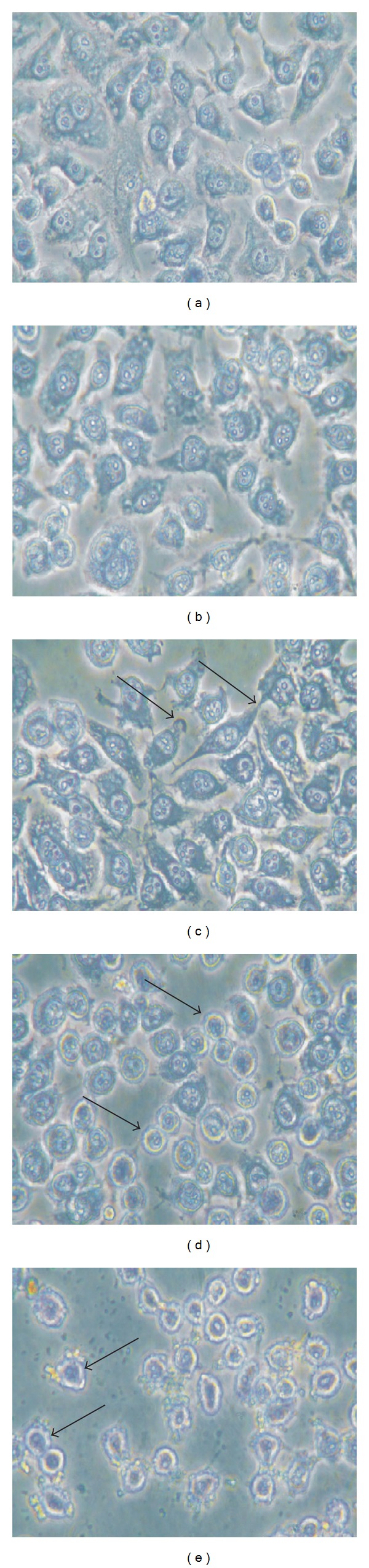
Phase contrast microscopic images of L-132 cells stained with Leishman's stain. The cells were cultured with (a) DMEM medium only (control), (b) 5 *μ*g/mL ZnO-NP, (c) 25 *μ*g/mL ZnO-NP, (d) 50 *μ*g/mL ZnO-NP, and (e) 100 *μ*g/mL ZnO-NP. Arrows indicate shrinkage and rounding of cells. The magnification was 200x.

The morphology of human lung epithelial cells was examined after ZnO-NPs exposure using phase contrast microscopy. Cells exposed to 50 nm ZnO-NPs for 24 h showed a round shape morphology than control cells (Figure 2). At higher concentration cells appeared to be strongly damaged with a shrunken morphology and detached from the substrate indicating an almost complete destruction of the cells exposed to ZnO-NPs (Figure 2). 

### 3.4. Effect of ZnO-NPs on ROS Production

**Figure 3:**
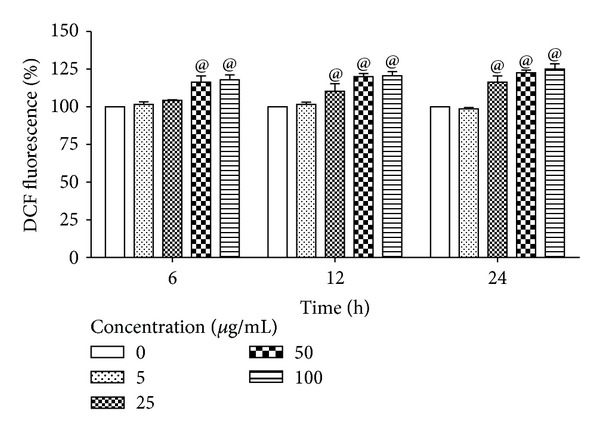
Time-dependant ROS generation by ZnO-NPs in L-132 cells. DCF-fluorescence intensity in L-132 cells after exposure to 5, 25, 50, and 100 *μ*g/mL of ZnO-NPs for different time periods, namely, 6, 12, and 24 h. Values were the mean ± SD from three independent experiments. Significance was indicated by  ^@^ *P* < 0.05 versus control.

The ability of 50 nm ZnO-NP to induce intracellular oxidant production in L-132 cells was assessed by measuring DCF fluorescence as a marker of ROS generation. With the increase of exposure time, a significant ROS formation was observed. ZnO-NPs significantly induced the formation of ROS from 16% to 25% at concentration of 25 *μ*g/mL to 100 *μ*g/mL after 24 h of exposure (Figure 3). 

### 3.5. Effect of ZnO-NPs on Intracellular GSH Levels

**Figure 4:**
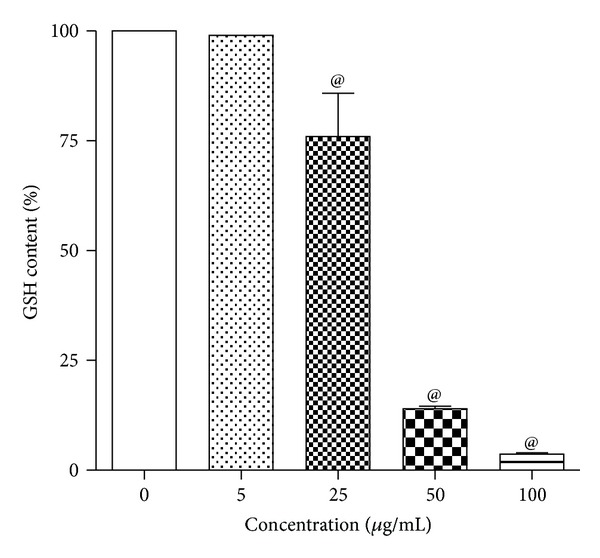
Effect of ZnO-NPs on GSH levels in L-132 cells. Cells were exposed to different concentrations of ZnO-NPs for 24 h. At the end of the exposure, cells were washed with PBS, and GSH levels were measured. Control cells cultured in ZnO-free medium were run in parallel to the exposed groups. Values were the mean ± SD from three independent experiments. Significance was indicated by ^@^ *P* < 0.05 versus control.

The intracellular GSH measurement has shown decrease in the intracellular GSH level with the increasing concentrations of ZnO-NPs after 24 h exposure. The significant difference in intracellular GSH level was observed at 50 and 100 *μ*g/mL with remaining GSH of about 14% and 4%, respectively, compared to control (Figure 4).

### 3.6. Effect of ZnO-NPs on DNA

**Figure 5:**
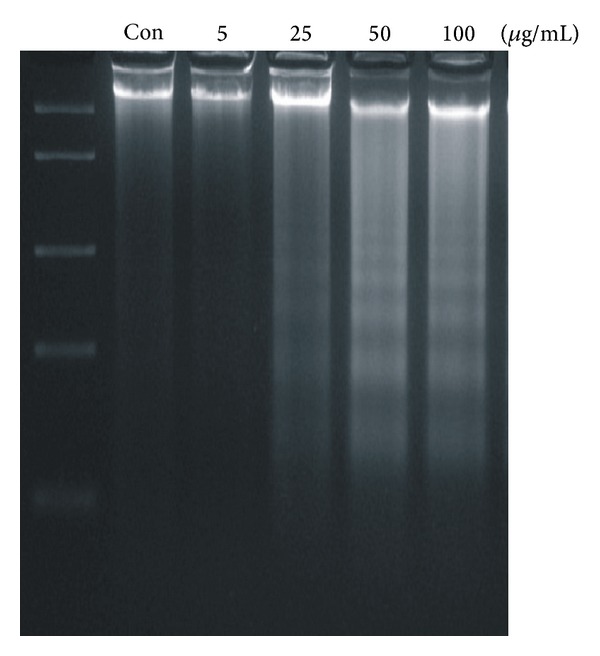
DNA fragments of L-132 cells on exposure to ZnO-NP for 48 h.

**Figure 6:**
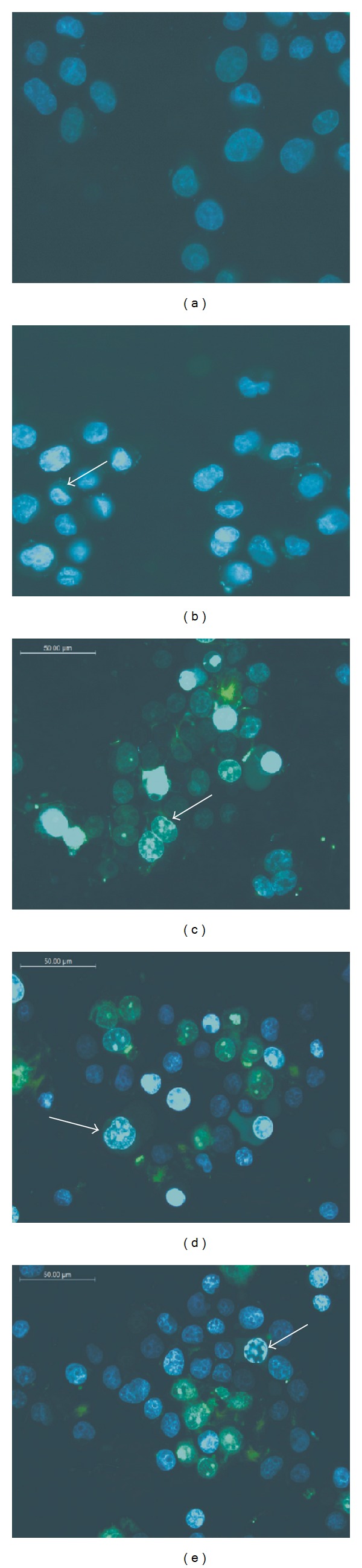
Fluorescent microscopic images of L-132 cells stained with Hoechst. The cells were cultured with (a) DMEM medium only (control), (b) 5 *μ*g/mL ZnO-NP, (c) 25 *μ*g/mL ZnO-NP, (d) 50 *μ*g/mL ZnO-NP, and (e) 100 *μ*g/mL ZnO-NP. Arrows indicate nuclear condensation and apoptotic body formation. The magnification was 400x.

L-132 cell exposed to 50 nm ZnO-NPs for 48 h showed DNA damage (Figure 5), while 24 h of exposure did not show any damage. Extended exposure of ZnO-NPs caused concentration-dependant DNA damage which was observed in the form of ladder. DNA damage was also observed by Hoechst staining. The nuclei of exposed cells appeared to be fragmented, smaller, and rougher, with condensed nuclear material (Figure 6).

### 3.7. Effect of ZnO-NPs on Metallothionein Gene Expression

**Figure 7:**
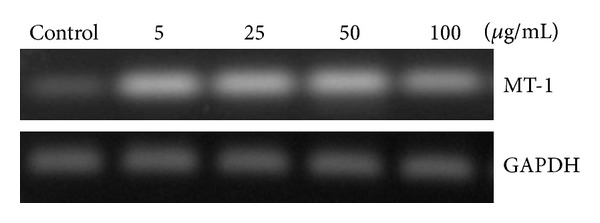
MT-1 mRNA expression from L-132 cells upon exposure to the different concentrations (5, 25, 50, and 100 *μ*g/mL) of ZnO-NPs, and GAPDH was used as housekeeping gene.

Metallothionein is known to facilitate metal detoxification and is involved in scavenging of free radicals [35]. After 24 h exposures of L-132 cells to 50 nm ZnO-NPs, upregulation in MT gene expression was observed. Although the expression was increased initially from concentration of 5 to 50 *μ*g/mL, later it decreased at 100 *μ*g/mL (Figure 7). 

## 4. Discussion

Although the beneficial effects of ZnO-NPs have attracted considerable attention in terms of nanomedicine [29, 36–38], potential biological and environmental hazards should also be taken into account. In the present study, 50 nm ZnO-NPs exposure significantly reduced cell viability of human lung cells starting at approximately 25 *μ*g/mL concentration. Cell viability data were further supported by the morphological studies. The lowering of cell density and the rounding of cells observed suggest that 25, 50, and 100 *μ*g/mL nanoparticle concentrations induced substantial cell death. The cytotoxicity results are in accordance with previous studies [16, 39–41]. 

Oxidative stress as a common mechanism for cell damage induced by nanoparticles is well documented. A wide range of nanomaterial species have been shown to generate ROS both *in vivo* and *in vitro* [2, 42–44]. Similarly, in the present study, 50 nm ZnO-NPs showed generation of ROS with significant depletion of reduced glutathione store. It is well-known that oxidative stress leads to cell death, either by apoptosis or necrosis depending on its extent of severity. Severe oxidative stress to cells causes necrosis while the moderate one causes apoptosis [45]. In the present study, the type of cell death (apoptosis/necrosis) after 50 nm ZnO-NPs exposures was evaluated by DNA damage analysis and Hoechst staining. DNA fragmentation is a characteristic feature of apoptosis [33]. Formation of the larger DNA fragments has been shown to occur in the absence of oligonucleosome formation [46]. Moreover, cleavage of DNA into the larger fragments is sufficient to allow chromatin condensation and subsequent apoptosis in the absence of oligonucleosome formation. In agreement to this, exposure of 50 nm ZnO-NPs produced large fragments of DNA in the range of 100 to 850 bp showing ladder formation in agarose gel. Hoechst staining also showed chromatin condensation and apoptotic body formation at higher concentration of ZnO-NPs, suggesting apoptotic type of cell death. Thus, it is possible that the ZnO-NPs might have induced apoptosis in human lung cells. 

Since metallothionein (MT) is considered as one of the essential biomarkers in metal-induced toxicity [47] facilitating metal detoxification and protection from free radicals [48], the ability of ZnO-NPs to modulate the expression of MT gene was also assessed. In present study, 50 nm ZnO-NP exposed cells showed upregulation of the MT gene. At lower concentrations of ZnO-NPs, increase in MT gene expression was observed which later decreased at higher concentrations. This finding is consistent with the results of biochemical and cytotoxicity data. MT gene expression study indicates that, during the cellular stress-induced by 50 nm ZnO-NPs, initially cells were able to protect themselves by upregulating MT gene using its antioxidant and metal detoxifying properties but at higher concentrations of ZnO-NPs; same phenomenon did not work because of greater extent of cell stress showing decrease in MT expression.

In summary, 50 nm ZnO-NPs induced cytotoxicity in cultured human lung epithelial cells (L-132) by elevating oxidative stress in a concentration-dependant fashion. ZnO-NPs also induced DNA damage characterized by chromatin condensation and DNA ladder pattern illustrating apoptotic type of cell death. Therefore, oxidative stress-induced apoptosis can be considered as one of the pathways of toxicity by ZnO-NPs. Hence, care has to be taken while processing and formulating the nanoparticles till its final finished product.

## Figures and Tables

**Table 1:** Particle characterization.

Particles	Description	Size using TEM^a^ (nm)	Size in media^b^ (nm)	PDI^c^	Zeta potential^d^ (mV)
ZnO-NP	Zinc oxide nanopowder	50.24 ± 8.19	93.6 ± 13.95	0.270 ± 0.042	−17.81 ± 5.63

^a^Using transmission electron microscopy.

^b, c, and d^Using Zeta PALS.

PDI: polydispersity index.
